# Association of Population Well-Being With Cardiovascular Outcomes

**DOI:** 10.1001/jamanetworkopen.2023.21740

**Published:** 2023-07-05

**Authors:** Erica S. Spatz, Brita Roy, Carley Riley, Dan Witters, Jeph Herrin

**Affiliations:** 1 Section of Cardiovascular Medicine, Department of Medicine, Yale School of Medicine, New Haven, Connecticut; 2 Yale University/Yale New Haven Health Center for Outcomes Research and Evaluation, New Haven, Connecticut; 3 Section of General Internal Medicine, Department of Medicine, Yale School of Medicine, New Haven, Connecticut; 4 Department of Pediatrics, University of Cincinnati College of Medicine, Cincinnati, Ohio; 5 Division of Critical Care, Cincinnati Children’s Hospital Medical Center, Cincinnati, Ohio; 6 Gallup National Health and Well-Being Index, Omaha, Nebraska

## Abstract

**Importance:**

Mortality from cardiovascular disease (CVD) varies across communities and is associated with known structural and population health factors. Still, a population’s well-being, including sense of purpose, social relationships, financial security, and relationship to community, may be an important target to improve cardiovascular health.

**Objective:**

To examine the association of population level measures of well-being with rates of CVD mortality in the US.

**Design, Setting, and Participants:**

This cross-sectional study linked data from the Gallup National Health and Well-Being Index (WBI) survey to county-level rates of CVD mortality from the Centers for Disease Control and Prevention Atlas of Heart Disease and Stroke. Participants were respondents of the WBI survey, which was conducted by Gallup with randomly selected adults aged 18 years or older from 2015 to 2017. Data were analyzed from August 2022 to May 2023.

**Main Outcomes and Measures:**

The primary outcome was the county-level rate of total CVD mortality; secondary outcomes were mortality rates for stroke, heart failure, coronary heart disease, acute myocardial infarction, and total heart disease. The association of population well-being (measured using a modified version of the WBI) with CVD mortality was assessed, and an analysis of whether the association was modified by county structural factors (Area Deprivation Index [ADI], income inequality, and urbanicity) and population health factors (percentages of the adult population who had hypertension, diabetes, or obesity; were currently smoking; and were physically inactive) was conducted. Population WBI and its ability to mediate the association of structural factors associated with CVD using structural equation models was also assessed.

**Results:**

Well-being surveys were completed by 514 971 individuals (mean [SD] age 54.0 [19.2] years; 251 691 [48.9%] women; 379 521 [76.0%] White respondents) living in 3228 counties. Mortality rates for CVD decreased from a mean of 499.7 (range, 174.2-974.7) deaths per 100 000 persons in counties with the lowest quintile of population well-being to 438.6 (range, 110.1-850.4) deaths per 100 000 persons in counties with the highest quintile of population well-being. Secondary outcomes showed similar patterns. In the unadjusted model, the effect size (SE) of WBI on CVD mortality was −15.5 (1.5; *P* < .001), or a decrease of 15 deaths per 100 000 persons for each 1-point increase of population well-being. After adjusting for structural factors and structural plus population health factors, the association was attenuated but still significant, with an effect size (SE) of −7.3 (1.6; *P* < .001); for each 1-point increase in well-being, the total cardiovascular death rate decreased by 7.3 deaths per 100 000 persons. Secondary outcomes showed similar patterns, with mortality due to coronary heart disease and heart failure being significant in fully adjusted models. In mediation analyses, associations of income inequality and ADI with CVD mortality were all partly mediated by the modified population WBI.

**Conclusions and Relevance:**

In this cross-sectional study assessing the association of well-being and cardiovascular outcomes, higher well-being, a measurable, modifiable, and meaningful outcome, was associated with lower CVD mortality, even after controlling for structural and cardiovascular-related population health factors, indicating that well-being may be a focus for advancing cardiovascular health.

## Key Points

**Question:**

Is population well-being (a comprehensive and validated metric consisting of having a successful life career, social relationships, financial security, relationship to community, and good physical health) associated with cardiovascular disease (CVD) mortality?

**Findings:**

In this cross-sectional study of 514 971 individuals, population well-being was associated with CVD mortality, independently of structural factors (eg, socioeconomic status) and population health metrics (eg, population rates of hypertension, diabetes, obesity, and physical inactivity). Additionally, population well-being mediated the association of structural factors with CVD mortality.

**Meaning:**

These findings suggest that population well-being is a measurable and modifiable metric that may offer a focus of immediate intervention to improve cardiovascular health outcomes.

## Teaser

This cross-sectional study assesses population well-being, a comprehensive and validated metric, and its association with cardiovascular disease mortality among adults in the US.

## Introduction

The incidence of disease and premature death from myocardial infarction, stroke, and congestive heart failure varies across the US, and traditional factors associated with the risk for cardiovascular disease (CVD) (eg, hypertension, hyperlipidemia, and diabetes) only partially explain this variation.^1,2,3,4^ Cardiovascular outcomes are impacted by larger societal aspects of a population,^5,6^ including the physical and social environment and economic opportunities, all of which play a role in determining health behaviors, positive and negative psychological health states, social connectedness, access to health care, and quality of health care. Therefore, as health systems aim to improve population health, there is growing interest in upstream, holistic measures that capture the physical, social, and psychological health of individuals. Toward this goal, measures have been developed, validated, and used to assess both individual and population well-being.^7,8^ These measures have been used in prior research to show strong associations of population well-being with health outcomes,^9^ lower rates of hospitalizations (including cardiovascular hospitalizations), emergency department and hospital utilization, and greater use of preventative services.^10,11,12,13,14,15,16^

Conceptually, associations of population well-being with cardiovascular health outcomes may reflect community-level attributes such as access to basic health care and social services, safe and clean streets, public transportation, green spaces, and healthy foods, as well as social aspects such as neighborhood cohesion, generosity among people, and a respectful working environment.^17,18^ Living in a community with high well-being may buffer the negative impact of stressful events and reduce the risk for illness and disease throughout the life course. Importantly, although well-being is associated with economic status, which can be challenging to address from a health system lens, well-being is also mediated by other factors in the community that are modifiable through community engagement and action in combination with public policy.^19^ Previous work^20^ has identified several high well-being counties that are among the counties with the highest poverty levels in the US. During a time of social upheaval and greater awareness of how social determinants of health and structural racism lead to health disparities, population well-being may offer a focus of immediate intervention to improve equity in cardiovascular health outcomes.^21^ More data demonstrating an association of well-being with cardiovascular outcomes could support this approach.

Therefore, we sought to examine the county-level association of population well-being, a component of population health, with CVD mortality. Using results from the Gallup National Health and Well-Being Index (WBI)^22^ (previously known as the Gallup-Sharecare Well-Being Index), a comprehensive, validated, multidimensional assessment of well-being, we compared area-level well-being with rates of total cardiovascular mortality, adjusting for structural factors (eg, poverty, income inequality, and rurality) and population health metrics (eg, population hypertension and diabetes rates). We also identified elements of well-being most associated with CVD mortality and examined the extent to which well-being mediates the association of structural and population health factors with CVD mortality.

## Methods

### Overview

This cross-sectional, observational study was approved by the institutional review board of Yale University and follows the Strengthening the Reporting of Observational Studies in Epidemiology (STROBE) reporting guideline. Informed consent was not required because the data were from anonymous surveys previously collected by Gallup in accordance with 45 CFR §46. Area-level measures of well-being and cardiovascular mortality were assessed for association across the US. Our unit of observation was the Federal Information Processing System (FIPS) code, which corresponds to a US county or county equivalent (eg, borough, parish, or township). We used a modified version of the WBI and applied weighted regression models to assess associations.

### Data

#### Well-Being

Our primary independent variable, an area measure of well-being, was constructed using data from the Gallup WBI collected from 2015 to 2017. This index includes 5 elements of well-being (having a successful life career, social relationships, financial security, relationship to community, and good physical health) and is scored on a scale of 0 to 100, with 0 being the lowest well-being and 100 being the highest well-being. The survey was developed on the basis of foundational work by Gallup from millions of participants, and has been demonstrated to be comprehensive, psychometrically valid, and reliable in over 13 000 individuals from 3 independent cohorts.^8^ Life evaluation is additionally assessed by Gallup using the Cantril Self-Anchoring Scale,^23^ which asks individuals about perceptions of their life now (current life satisfaction) and in 5 years (future life optimism) in relation to their best life on a scale of 0 to 10; both the current life satisfaction measurement and future life optimism measurement are included in the overall WBI, but not as part of the 5 elements of well-being.

For the WBI, Gallup surveys individuals who are 18 years or older by phone. Landline and cellular telephone numbers are selected using random−digit dialing methods. In the case of landline surveys, the adult with the most recent birthday is selected to represent a household. Surveys are conducted in both English and Spanish. More than 175 000 surveys are conducted each year, with weights assigned to final samples to match the US population according to sex, age, race, Hispanic ethnicity, education, region, population density, and phone status (cellular telephone only, landline only, both cellular telephone and landline, and mostly cellular telephone). Each national sample of adults in 2015 and 2016 included a minimum quota of 60% cellphone respondents and the 2017 sample included a minimum quota of 70% cellphone respondents. Additional minimum quotas were by time zone within a region.

For our primary outcome, we constructed a modified version of the WBI in which the physical health element was omitted. In secondary analyses, we examined the separate elements (other than physical health) of the WBI. Individual respondents were mapped to FIPS codes using their telephone number; we took the mean of each modified WBI and element score over each FIPS code to produce our final set of independent measures.

#### Cardiovascular Mortality

For measures of cardiovascular mortality, we used data from the Centers for Disease Control and Prevention Interactive Atlas of Heart Disease and Stroke,^24^ which is age and sex adjusted by FIPS code for the US. We used estimates from 2016 to 2018 to align with our well-being data. Our primary outcome was total CVD mortality; secondary outcomes were stroke, heart failure, coronary heart disease, heart attack, and all heart disease mortality rates.

#### Structural and Health Factors

We adjusted our results for structural factors and population health metrics that have previously been demonstrated to be associated with cardiovascular outcomes, and as such, might modify the association of well-being with CVD mortality. Structural factors were assessed using the Area Deprivation Index (ADI), which includes measures of education, employment, housing quality, and poverty. The ADI is derived from the American Community Survey US Census data and previously has been demonstrated to be associated with health outcomes.^25,26^ We also included a measure of income inequality (the Gini Index), as well as a measure of urbanicity because of research indicating that living in more populated areas is associated with higher well-being than living in less populated areas.^27^ For urbanicity we used the National Center for Health Statistics urban classification codes.^28^ FIPS area data were mapped as nonmetropolitan area, medium or small metropolitan area, large fringe metropolitan area, and large central metropolitan area. Population health metrics associated with CVD mortality also came from the Centers for Disease Control and Prevention Interactive Atlas of Heart Disease and Stroke^24^ and included the percentage of the adult population who had hypertension, diabetes, and obesity, who were currently smoking, and who were physically inactive.

### Statistical Analysis

We summarized WBI and modified WBI scales by county characteristics, using quintiles for continuous factors, including structural factors captured with the ADI. To assess the association of well-being with CVD mortality rates, we first graphed county-level CVD mortality rates against our modified WBI, and summarized disease-specific mortality rates over quintiles of the modified WBI.

Then, to test for associations and to assess the magnitude of the associations, we estimated a series of weighted linear regression models, with each observation weighted for number of survey responses. In a first set of models (1 for each outcome), we included only the modified WBI or 1 of the elements as an independent variable. In the second set, we adjusted for the Gini index, urbanicity, and the ADI. In the third set of models, we additionally adjusted for rates of diabetes, obesity, hypertension, smoking, and physical inactivity.

Then, to assess which components of well-being are most associated with CVD mortality, we estimated for each outcome a separate model that included the elements of well-being, as well as the 2 life evaluation scores. These models were all adjusted for all structural and health measures.

Finally, we conducted path analyses to formally assess whether population WBI mediates the association of structural factors with CVD. Mediation analysis is used to assess whether and how much a given factor mediates the association of 2 other factors.^29,30^ We assessed whether the modified WBI mediated the association of each of these structural factors with total CVD mortality, and if so, how much of the association of the structural factor with mortality was mediated by the association of the structural factor with well-being. Specifically, we estimated separate structural equation models, one for each structural factor, to decompose the association of the factor with CVD mortality into a direct association and an indirect (that is, through the association with WBI) association; we report the direct and indirect associations and mediation effect sizes from each model.

Given that area mediation effects may be larger than a single county, there is the potential for spatial correlation of errors. We therefore performed several secondary analyses to determine whether there was spatial correlation and to assess the potential impact on our main findings. For each county, we obtained the coordinates of the population centrum based on the 2010 US Census^31^ and constructed a spatial weighting matrix reflecting the inverse distance (in kilometers) between each centrum. We estimated for each outcome an empty model with WBI as the independent variable and performed the Moran test for spatial independence using the weight matrix. Since all tests indicated spatial correlation, we replicated the main analyses using spatial autoregressive random effects models.^32^ These models were also weighted for population, but we treated them as secondary because we did not use multiple imputation to account for missing data due to the technical challenges of doing so.

For all models we report the coefficients, SE, and *P* value; 2-sided *P* < .05 was taken to be significant. All analyses were done using Stata statistical software version 17.0 (StataCorp). Missing values were accounted for using multiple chained imputation with 20 imputation sets. Data analysis was conducted from August 2022 to May 2023.

## Results

From 2015 through 2017, Gallup National Health and WBI surveys were conducted among 514 971 participants (mean [SD] age 54.0 [19.2] years; 251 691 [48.9%] women; 379 521 [76.0%] White respondents) living in 3228 different US counties or county equivalents. The number of surveys per county ranged from 1 to 10 827. Table 1 shows the mean county-level WBI and modified WBI scores by several community attributes, stratified into quintiles. We observed marginally but statistically significant lower well-being scores among counties with greater income inequality and more area deprivation. Counties with higher rates of hypertension, diabetes, physical inactivity, obesity, and smoking rates had marginally but significantly lower well-being scores.

**Table 1.** Description of Counties by Size, Geography, Demographics, and Poverty by Quintiles of Total WBI

Characteristic	Respondents, No. (%) (N = 3228)	WBI		WBI modified	
		Mean (SD)	*P* value	Mean (SD)	*P* value
Overall score	NA	62.7 (3.4)	NA	47.0 (3.0)	NA
Structural factors					
Urbanicity					
Large central metropolitan area	68 (2.1)	62.5 (1.5)	<.001	45.7 (1.4)	<.001
Large fringe metropolitan area	368 (11.4)	63.0 (2.1)		47.0 (1.9)	
Medium or small metropolitan area	730 (22.6)	62.7 (2.6)		46.8 (2.2)	
Rural area	1974 (61.2)	62.6 (3.9)		47.0 (3.4)	
Missing	88 (2.7)	57.9 (1.2)		45.8 (6.1)	
Income inequality (Gini Index), quintile					
1	674 (20.9)	63.1 (3.5)	<.001	47.2 (3.2)	<.001
2	647 (20.0)	62.9 (3.0)		47.3 (2.7)	
3	654 (20.3)	62.6 (3.1)		46.9 (2.7)	
4	614 (19.0)	62.4 (3.5)		46.8 (3.1)	
5	631 (19.5)	62.2 (3.7)		46.6 (3.1)	
Missing	8 (0.2)	57.9 (1.2)		45.8 (6.1)	
Area Deprivation Index, quintile					
1	654 (20.3)	63.9 (2.4)	<.001	47.5 (2.1)	<.001
2	632 (19.6)	63.1 (2.9)		47.2 (2.7)	
3	622 (19.3)	62.7 (2.9)		47.1 (2.7)	
4	644 (20.0)	61.9 (3.6)		46.7 (3.2)	
5	621 (19.3)	61.3 (4.2)		46.1 (3.8)	
Missing	46 (1.4)	64.3 (5.4)		48.2 (4.9)	
Population health metrics					
Hypertension, quintile					
1	646 (20.0)	63.6 (2.3)	<.001	47.2 (2.0)	.001
2	637 (19.7)	63.2 (3.0)		47.3 (2.8)	
3	613 (19.0)	62.7 (3.5)		47.0 (3.2)	
4	622 (19.3)	62.3 (3.7)		46.9 (3.3)	
5	624 (19.3)	61.4 (3.8)		46.4 (3.3)	
Missing	86 (2.7)	57.9 (1.2)		45.8 (6.1)	
Diabetes, quintile					
1	674 (20.9)	63.7 (4.1)	<.001	47.6 (3.7)	
2	615 (19.1)	63.2 (3.2)		47.3 (2.9)	<.001
3	644 (20.0)	62.7 (2.8)		47.0 (2.6)	
4	643 (19.9)	62.0 (3.0)		46.5 (2.7)	
5	644 (20.0)	61.5 (3.3)		46.3 (2.8)	
Missing	8 (0.2)	57.9 (1.2)		45.8 (6.1)	
Obesity, quintile					
1	634 (19.6)	63.8 (4.0)	<.001	47.4 (3.7)	<.001
2	638 (19.8)	63.1 (2.9)		47.3 (2.7)	
3	629 (19.5)	62.7 (3.0)		47.1 (2.7)	
4	622 (19.3)	62.0 (3.2)		46.5 (2.8)	
5	619 (19.2)	61.6 (3.3)		46.4 (2.9)	
Missing	86 (2.7)	57.9 (1.2)		45.8 (6.1)	
Physical inactivity, quintile					
1	629 (19.5)	64.1 (3.3)	<.001	47.6 (3.0)	<.001
2	642 (19.9)	63.3 (3.4)		47.3 (3.1)	
3	632 (19.6)	62.6 (3.1)		47.0 (2.8)	
4	611 (18.9)	62.1 (3.1)		46.7 (2.8)	
5	628 (19.5)	61.2 (3.4)		46.1 (3.0)	
Missing	86 (2.7)	57.9 (1.2)		45.8 (6.1)	
Smoking, quintile					
1	649 (20.1)	64.2 (3.4)	<.001	47.7 (3.3)	<.001
2	619 (19.2)	63.6 (2.9)		47.6 (2.8)	
3	639 (19.8)	62.6 (3.0)		47.0 (2.8)	
4	617 (19.1)	61.8 (3.0)		46.5 (2.6)	
5	618 (19.1)	60.9 (3.6)		46.0 (3.1)	
Missing	86 (2.7)	57.9 (1.2)		45.8 (6.1)	

Abbreviations: NA, not applicable; WBI, Well-Being Index.

The Figure shows the inverse association of population well-being with CVD mortality rate. Among all counties (Table 2), the CVD mortality rate was 462.0 deaths per 100 000 persons. Mortality rates for CVD decreased from a mean of 499.7 (range, 174.2-974.7) deaths per 100 000 persons in counties with the lowest quartile of population well-being to a mean of 438.6 (range, 110.1-850.4) deaths per 100 000 persons in counties with the highest quartile of population well-being, resulting in a difference of 61.1 deaths per 100 000 persons between the lowest and highest WBI counties. The crude rates for CVD, stroke, heart failure, coronary heart disease, heart attack, and all heart disease mortality decreased as population well-being increased.

**Figure.**
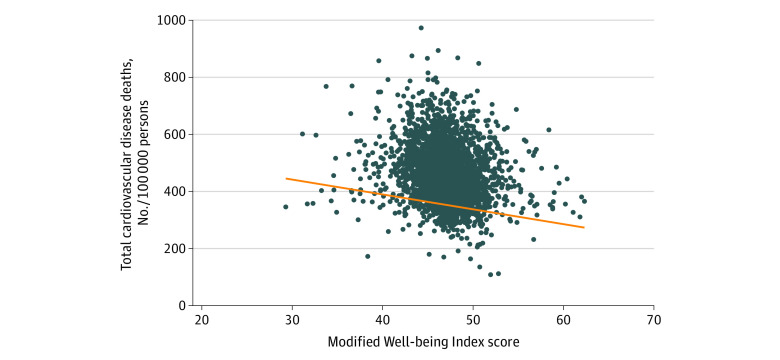
Scatter Plot of County Cardiovascular Disease Mortality Rate vs Modified Well-Being Index The scatter plot shows modified Well-Being Index scores according to county-level cardiovascular disease mortality rate per 100 000 persons.

**Table 2.** Summary of Cardiovascular Disease Mortality Rates (Per 100 000 Persons) for All Counties and by Q of Overall Modified WBI

Mortality	Mortality rate, mean (range) deaths per 100 000 persons					
	All counties	Modified WBI Q1	Modified WBI Q4	Modified WBI Q4	Modified WBI Q4	Modified WBI Q5
Total cardiovascular disease^a^	462.0 (110.1-974.7)	499.7 (174.2-974.7)	472.1 (181.5-895.6)	455.6 (171.8-757.5)	444.1 (193.2-869.7)	438.6 (110.1-850.4)
Stroke	76.1 (0.0-172.2)	78.8 (0.0-172.2)	77.5 (30.7-166.6)	76.4 (2.0-163.1)	74.0 (23.6-164.9)	73.6 (25.7-136.8)
Heart failure	214.3 (51.2-533.4)	226.8 (81.3-439.3)	217.3 (77.1-533.4)	208.0 (66.2-430.4)	210.4 (62.8-466.6)	208.7 (51.2-428.9)
Coronary heart disease	196.5 (17.0-590.3)	219.0 (89.6-590.3)	199.2 (64.1-449.0)	191.0 (17.0-444.9)	185.3 (60.7-431.7)	188.1 (40.1-502.9)
Heart attack	76.7 (0.0-501.3)	90.1 (0.0-501.3)	75.4 (14.4-355.3)	70.8 (0.0-291.3)	71.1 (11.0-338.3)	76.1 (6.5-422.1)
All heart disease	354.9 (83.4-810.5)	387.1 (174.2-735.0)	363.0 (132.7-771.3)	347.7 (155.2-666.5)	340.4 (126.3-810.5)	336.2 (83.4-700.6)

Abbreviations: Q, quintile; WBI, Well-Being Index.

^a^ Components of cardiovascular disease mortality do not sum to total cardiovascular disease mortality due to suppression of data for counties with few events.

Table 3 shows the results of 3 models of association with well-being and each of its components, and each cardiovascular outcome. In the unadjusted model (no covariates), the effect size (SE) of WBI on CVD mortality was −15.5 (1.5; *P* < .001), indicating that total cardiovascular deaths decreased by 15.5 deaths per 100 000 persons for each 1-point increase of population well-being. This association was mostly mediated by deaths from all heart disease, and less from deaths from stroke. After adjusting for structural factors (model 2) and structural plus population health factors (model 3), the association was attenuated but still significant; for each 1-point increase in well-being, the total cardiovascular death rate decreased by a mean (SE) of 7.3 (1.6) per 100 000 persons (*P* < .001). All components of well-being were inversely associated with total cardiovascular death except anticipated life satisfaction. The 6 fully adjusted models for well-being are reported in eTable 1 in Supplement 1. In the analysis using spatial autoregressive models (eTable 2 in Supplement 1) results were similar, though slightly attenuated. In the secondary analysis of well-being elements, only the community and financial well-being elements were independently associated with CVD mortality (Table 4).

**Table 3.** Association of Modified WBI, Index Elements, and Life Evaluation Scores With Cardiovascular Outcomes

Outcome and model^a^	Coefficient (SE)						
	Overall WBI modified score	Modified WBI element score				Life evaluation score	
		Community	Financial	Purpose	Social	CLS	FLO
Total cardiovascular disease mortality							
1	−15.5 (1.5)^b^	−7.2 (0.6)^b^	−10.3 (1.0)^b^	−2.2 (1.7)	−8.5 (1.7)^b^	−1.5 (0.1)^b^	−0.7 (0.1)^b^
2	−13.3 (1.5)^b^	−6.1 (0.5)^b^	−7.5 (0.9)^b^	−5.8 (1.5)^b^	−5.1 (1.5)^c^	−0.9 (0.1)^b^	−0.2 (0.1)^c^
3	−7.3 (1.6)^b^	−3.1 (0.5)^b^	−3.4 (0.9)^b^	−2.7 (1.4)	−3.0 (1.3)^c^	−0.4 (0.1)^c^	−0.0 (0.1)
Stroke mortality							
1	−0.7 (0.3)^c^	−0.6 (0.2)^c^	−0.8 (0.2)^b^	1.2 (0.3)^b^	0.2 (0.3)	−0.1 (0.0)	−0.0 (0.0)
2	−0.1 (0.3)	−0.2 (0.2)	−0.4 (0.2)	0.8 (0.2)^b^	0.6 (0.3)^c^	0.0 (0.0)	0.1 (0.0)^c^
3	0.2 (0.3)	−0.0 (0.2)	−0.2 (0.2)	1.0 (0.2)^b^	0.7 (0.3)^c^	0.1 (0.0)	0.1 (0.0)^b^
Heart failure mortality							
1	−0.9 (0.8)	0.2 (0.3)	−0.9 (0.5)^c^	0.8 (0.7)	−3.6 (0.6)^b^	−0.5 (0.1)^b^	−0.6 (0.1)^b^
2	−2.5 (0.7)^c^	−0.7 (0.3)^c^	−0.6 (0.4)	−1.9 (0.6)^c^	−2.1 (0.5)^b^	−0.2 (0.1)^c^	−0.1 (0.0)^b^
3	−1.3 (0.8)	−0.1 (0.3)	−0.4 (0.4)	−1.2 (0.6)	−1.3 (0.6)^c^	−0.1 (0.1)	−0.1 (0.0)^c^
Coronary heart disease mortality							
1	−9.4 (1.7)^b^	−3.9 (0.4)^b^	−5.9 (0.8)^b^	−3.3 (1.7)	−5.5 (1.7)^c^	−0.9 (0.1)^b^	−0.5 (0.1)^b^
2	−8.8 (1.8)^b^	−3.9 (0.4)^b^	−4.9 (0.9)^b^	−4.6 (1.7)^c^	−4.0 (1.7)^c^	−0.7 (0.1)^b^	−0.3 (0.1)^c^
3	−5.2 (2.0)^c^	−2.2 (0.5)^b^	−2.4 (0.9)^c^	−2.6 (1.7)	−2.7 (1.6)	−0.4 (0.2)^c^	−0.2 (0.1)^c^
Heart attack mortality							
1	−1.4 (0.4)^b^	−0.3 (0.2)	−1.8 (0.3)^b^	0.7 (0.3)^c^	−2.0 (0.4)^b^	−0.3 (0.0)^b^	−0.3 (0.0)^b^
2	−1.6 (0.4)^b^	−0.6 (0.2)^c^	−1.2 (0.3)^b^	−0.6 (0.3)^c^	−1.0 (0.4)^c^	−0.1 (0.0)^b^	−0.1 (0.0)^c^
3	−0.2 (0.5)	0.1 (0.2)	−0.3 (0.3)	0.0 (0.3)	−0.6 (0.4)	−0.0 (0.0)	−0.1 (0.0)^c^
All heart disease mortality							
1	−13.5 (1.4)^b^	−5.8 (0.4)^b^	−8.8 (0.8)^b^	−3.0 (1.5)	−8.0 (1.6)^b^	−1.3 (0.1)^b^	−0.7 (0.1)^b^
2	−11.9 (1.5)^b^	−5.1 (0.4)^b^	−6.6 (0.8)^b^	−6.1 (1.4)^b^	−5.2 (1.6)^c^	−0.9 (0.1)^b^	−0.3 (0.1)^c^
3	−6.6 (1.5)^b^	−2.5 (0.4)^b^	−3.0 (0.8)^c^	−3.3 (1.4)^c^	−3.3 (1.3)^c^	−0.4 (0.1)^c^	−0.1 (0.1)

Abbreviations: CLS, current life satisfaction; FLO, future life optimism; WBI, Well-Being Index.

^a^ Model 1 was unadjusted. Model 2 was adjusted for urbanicity, income inequality, and poverty quintile (defined as less than a high school diploma, or less than a college degree). Model 3 was adjusted for urbanicity, income inequality, poverty, diabetes quintile, obesity quintile, hypertension quintile, and smoking quintile.

^b^ Indicates *P* < .001.

^c^ Indicates *P* < .05.

**Table 4.** Association of Cardiovascular Disease Outcomes with WBI Elements and Life Evaluation Scores

Mortality^a^	Coefficient (SE)					
	WBI element				Life evaluation score	
	Community	Financial	Purpose	Social	Current life satisfaction	Future life optimism
Total cardiovascular disease	−1.8 (0.6)^b^	−2.0 (0.7)^b^	1.4 (1.2)	−1.0 (1.1)	−0.1 (0.1)	0.1 (0.1)
Stroke	−0.3 (0.1)^b^	−0.8 (0.2)^c^	1.6 (0.5)^b^	−0.0 (0.4)	0.0 (0.0)	0.1 (0.0)
Heart failure	0.2 (0.4)	−0.6 (0.4)	0.6 (0.7)	−1.0 (0.5)	0.1 (0.1)	−0.2 (0.0)^b^
Coronary heart disease	−0.7 (0.7)	0.3 (0.5)	−1.8 (1.3)	−0.2 (0.8)	−0.2 (0.1)	−0.1 (0.1)
Heart attack	0.4 (0.2)	0.1 (0.3)	0.2 (0.5)	−1.0 (0.4)^b^	0.1 (0.1)	−0.1 (0.0)^c^
All heart disease	−0.9 (0.6)	−1.5 (0.6)^b^	−0.1 (1.1)	−1.0 (0.9)	−0.1 (0.1)	0.0 (0.1)

Abbreviation: WBI, Well-Being Index.

^a^ Adjusted for income inequality, urbanicity, Area Deprivation Index, diabetes quintile, obesity quintile, and physical inactivity quintile.

^b^ Indicates *P* < .05.

^c^ Indicates *P* < .001.

Finally, mediation analyses (eTable 3 in Supplement 1) demonstrated that the associations of Gini Index and ADI with CVD mortality were both partly mediated by the modified population WBI. For example, as the Gini Index increased from 1 to 10, the number of CVD deaths increased by 7.3 deaths per 100 000 persons, of which approximately 6.9 of this was a direct mediation effect whereas the remainder was associated with the lower well-being that follows from poverty.

## Discussion

In this cross-sectional study of population well-being and cardiovascular outcomes, we found that higher well-being was associated with lower CVD mortality, even after controlling for structural and cardiovascular-related population health factors at the county level. We used a modified measure of well-being that excluded all physical health information, demonstrating that areas with higher measures of community, social, financial, and purpose well-being, and with greater life satisfaction, had lower rates of cardiovascular death. This association held for each well-being element, and for 5 types of CVD mortality, although the primary associations were with death from heart disease. The most significant elements of well-being were financial and community health; the social well-being element along with future life optimism were modestly, but independently, associated with all cardiovascular outcomes. We also found that population well-being significantly, although modestly, mediated the association of many structural risk factors with CVD mortality; the associations of the ADI and income inequality were all partly mediated by the association of higher well-being.

We know that CVD mortality is associated with individual level socioeconomic status, cardiometabolic risk factors, and positive and negative psychological attributes.^33,34,35^ However, these individual level factors do not account for all of the variation in cardiovascular disease outcomes in a population.^36^ In seminal work by Diez Roux et al,^37^ neighborhood deprivation was associated with incident coronary heart disease, even after controlling for personal income, education, and employment. Social determinants of health, including housing stability, quality, safety, affordability, and accessibility, as well as neighborhood environment, were also associated with cardiovascular outcomes and well-being.^38,39^ A 2014 study,^40^ which measured state-level structural racism using political participation, employment and job status, educational attainment, and judicial treatment as indicators, found an association of structural racism with rates of myocardial infarction among Black residents, but not White residents. Focusing on structural factors like poverty, education, and racism is critically important, and there is also a need for more comprehensive constructs of healthy communities that incorporate positive aspects such as life satisfaction, optimism, happiness, and thriving. A broader view of the population’s total well-being that is not merely the absence of negative factors but also the facilitation of positive factors, can open up opportunities for communities to improve cardiovascular outcomes through the promotion of well-being.^41,42^

Our findings suggest that interventions targeting increased well-being could have a significant association with CVD mortality, which continues to be the leading cause of death in the US, accounting for 20% of all deaths.^43^ Results from our path analyses indicating that well-being mediates the association of structural risk factors with CVD mortality (ie, income inequality and the ADI) support this claim. Although the indirect associations of well-being with CVD mortality are small, when considered across multiple community risk factors, the attenuating impact of well-being could be meaningful. Taken together, these findings suggest that targeting interventions to improve well-being in communities with lower socioeconomic measures could be an effective way to mitigate the increased risk of CVD death in those communities.

A focus on well-being could be particularly important for addressing the widening socioeconomic and racial disparities in cardiovascular disease and mortality while promoting positive factors that contribute to healthy lifestyle behaviors and social cohesion.^4,33,42^ Specifically, there is an urgent need to address the environmental, social, and community contexts that contribute to disparities in lifestyle behaviors (eg, physical activity, healthy diets, obesity, and smoking rates) and negative psychological health (eg, stress and anger), which affect well-being and can lead to disparities in cardiovascular risk factors, myocardial infarction, stroke, heart failure, and maternal morbidity and mortality.^9,44,45^ Addressing discrimination and racism, associated not only with psychological health,^46^ but also an individual’s sense of belonging and trust (captured in the community well-being element of the WBI), can affect a community’s togetherness and resilience. Engendering social cohesion by supporting families and community organizations can build greater social capital^47^ (captured in the social element of the WBI), which can then lead to improved mental well-being, along with greater awareness and advocacy around health. Other aspects of well-being were captured in the purpose element of the WBI (eg, job satisfaction) and in the life evaluation items (eg, the perception of opportunity for upward mobility). There are numerous interventions that have been found to improve subjective well-being. In a recent systematic review, Sakuraya et al^48^ identified 39 randomized trials with a pooled standardized difference (SE) of 0.5 (0.1). A project relevant to our specific measure of well-being is the Blue Zones Project by Sharecare,^19^ a community-led, collective-impact initiative, aimed at improving health and well-being in a set of US communities. Interventions were multilevel and included new or enhanced policies, processes, and programs. The Life Evaluation Index using the Cantril Ladder was used to measure impact. In a before-and-after comparative analysis with US communities and with the nation, the intervention communities experienced improved Life Evaluation Index scores following the intervention.^19^

### Limitations

This study should be interpreted in the context of several limitations. First, our analysis assesses the associations of well-being with CVD mortality across multiple diverse US counties. These associations may be different within counties. Second, given how intertwined well-being is with traditional factors associated with the risk for cardiovascular disease mortality, it can be difficult to distill the association of well-being from obesity, smoking, physical inactivity, and depression, all of which are known health factors associated with CVD mortality and well-being. We omitted the physical health element from the total well-being score to isolate the nontraditional factors associated with risk of CVD mortality. Moreover, these traditional factors only account for some of the risk for cardiovascular disease; focusing on individual behaviors alone, without attention to larger community well-being, may not be sufficient for actualizing real change in cardiovascular outcomes and health equity. Third, these data were collected before the COVID-19 pandemic, which likely impacted the lived environment and disease risk for most of the US, potentially altering the associations observed here.

## Conclusions

In conclusion, well-being offers a comprehensive framework and measurable outcome that is associated with CVD mortality. The most significant elements of well-being were financial and community well-being. Although a population’s financial well-being can be hard to modify without broad societal changes, community, social, and purpose well-being, which reflect people’s sense of engagement and the quality of their lived experiences in their community, can be modified to achieve the mutually overlapping goals of well-being, health equity, and cardiovascular health.

## Notes

Supplement 1. **eTable 1.** Model Results for the Fully Adjusted Models **eTable 2.** Results of Spatial Correlation Analysis **eTable 3.** Results of Mediation Analyses

Supplement 2.
Data Sharing Statement
