# Supplementary material for: Association of Population Well-Being With Cardiovascular Outcomes
Source: JAMA Netw Open. 2023 Jul 5;6(7):e2321740. doi: 10.1001/jamanetworkopen.2023.21740 (PMC10323707; doi:10.1001/jamanetworkopen.2023.21740)
Supplement: Supplement 1. — eTable 1. Model Results for the Fully Adjusted Models eTable 2. Results of Spatial Correlation Analysis eTable 3. Results of Mediation Analyses [file jamanetwopen-e2321740-s001.pdf]

Supplemental Online Content

Spatz ES, Roy B, Riley C, Witters D, Herrin J. Association of population well-being with cardiovascular outcomes. *JAMA Netw Open*. 2023;6(7):e2321740. doi:10.1001/jamanetworkopen.2023.21740

eTable 1. Model Results for the Fully Adjusted Models

eTable 2. Results of Spatial Correlation Analysis

eTable 3. Results of Mediation Analyses

This supplemental material has been provided by the authors to give readers additional information about their work.

© 2023 Spatz ES et al. *JAMA Network Open.*

eTable 1. Model results for the fully adjusted models

Model results for the fully adjusted models in Table 3; using the modified WBI as the independent variable. Coefficients are in units of deaths per 100,000.

	TCD mortality	Stroke mortality	HF mortality	CHD mortality	AMI mortality	AHD mortality
WBI	-7.268*** (-10.84)	0.168 (0.93)	-1.590*** (-3.45)	-4.656*** (-8.98)	-0.187 (-0.55)	-6.526*** (-10.72)
Gini Coefficient	-0.632 (-1.87)	-0.198* (-2.17)	-2.307*** (-9.92)	-0.0167 (-0.06)	0.269 (1.56)	-0.415 (-1.35)
ADI	0.110 (1.41)	0.103*** (4.90)	0.449*** (8.37)	-0.241*** (-4.00)	0.0939* (2.36)	0.0659 (0.93)
% diabetes	1.493* (2.43)	0.450** (2.71)	0.700 (1.66)	-1.073* (-2.26)	0.160 (0.51)	0.447 (0.80)
% obesity	2.250*** (6.61)	0.633*** (6.89)	2.946*** (12.59)	-0.167 (-0.63)	0.449** (2.59)	1.280*** (4.14)
% inactivity	2.705*** (8.17)	-0.428*** (-4.78)	-2.114*** (-9.28)	3.490*** (13.63)	0.951*** (5.63)	3.137*** (10.43)
% HTN	3.302*** (9.55)	1.031*** (11.04)	-0.929*** (-3.91)	0.823** (3.08)	0.154 (0.87)	2.229*** (7.10)
% smoking	5.746*** (11.27)	0.0711 (0.52)	2.354*** (6.71)	3.789*** (9.61)	1.008*** (3.87)	5.544*** (11.98)
Urban	ref	ref	ref	ref	ref	ref
Fringe Metro	-20.23*** (-6.79)	-3.407*** (-4.23)	-0.101 (-0.05)	-10.12*** (-4.39)	2.203 (1.45)	-12.98*** (-4.80)
Medium/ Small Metro	-16.39*** (-5.76)	-5.489*** (-7.14)	10.36*** (5.29)	-4.073 (-1.85)	5.284*** (3.64)	-9.363*** (-3.62)
Rural	-23.22*** (-6.20)	-10.44*** (-10.32)	21.51*** (8.34)	4.374 (1.51)	22.30*** (11.66)	-12.02*** (-3.53)
Constant	443.3*** (11.12)	23.98* (2.23)	272.0*** (9.92)	251.6*** (8.16)	-21.05 (-1.03)	361.5*** (9.98)
N	3228	3228	3228	3228	3228	3228
R^2	0.585	0.244	0.434	0.354	0.294	0.550

© 2023 Spatz ES et al. *JAMA Network Open.*

eTable 2

Results of spatial correlation analysis.

Outcome	Model[+]	WB Modified Coeff (SE)	Community Coeff (SE)	Financial Coeff (SE)	Purpose Coeff (SE)	Social Coeff (SE)	CLS Coeff (SE)	FLO Coeff (SE)
Total Cardiovascular Disease	1	-14.0 (0.8)**	-6.5 (0.4)**	-10.0 (0.4)**	-1.0 (0.7)	-8.5 (0.7)**	-1.4 (0.1)**	-0.7 (0.0)**
	2	-12.8 (0.7)**	-6.1 (0.3)**	-7.6 (0.4)**	-4.9 (0.6)**	-5.8 (0.6)**	-0.9 (0.1)**	-0.2 (0.0)**
	3	-6.8 (0.7)**	-3.0 (0.3)**	-3.5 (0.4)**	-2.4 (0.5)**	-3.2 (0.5)**	-0.4 (0.1)**	-0.0 (0.0)
Stroke	1	-0.4 (0.2)*	-0.4 (0.1)**	-0.7 (0.1)**	1.5 (0.1)**	0.3 (0.1)*	-0.0 (0.0)*	-0.0 (0.0)
	2	0.1 (0.2)	-0.1 (0.1)	-0.4 (0.1)**	1.2 (0.1)**	0.7 (0.1)**	0.0 (0.0)*	0.1 (0.0)**
	3	0.3 (0.2)	-0.0 (0.1)	-0.3 (0.1)*	1.2 (0.1)**	0.7 (0.1)**	0.1 (0.0)**	0.1 (0.0)**
Heart Failure	1	-0.4 (0.5)	0.3 (0.2)	-0.9 (0.3)**	1.1 (0.4)*	-3.2 (0.4)**	-0.4 (0.0)**	-0.6 (0.0)**
	2	-2.5 (0.4)**	-0.7 (0.2)**	-0.8 (0.2)*	-1.8 (0.4)**	-2.1 (0.4)**	-0.2 (0.0)**	-0.2 (0.0)**
	3	-1.3 (0.5)*	-0.1 (0.2)	-0.6 (0.3)*	-1.1 (0.4)*	-1.3 (0.4)**	-0.1 (0.0)*	-0.2 (0.0)**
Coronary Heart Disease	1	-8.7 (0.5)**	-3.7 (0.2)**	-5.8 (0.3)**	-2.8 (0.4)**	-5.9 (0.4)**	-0.9 (0.0)**	-0.5 (0.0)**
	2	-8.5 (0.5)**	-3.8 (0.3)**	-4.8 (0.3)**	-4.6 (0.4)**	-4.7 (0.4)**	-0.7 (0.0)**	-0.3 (0.0)**
	3	-4.7 (0.5)**	-2.0 (0.3)**	-2.1 (0.3)**	-2.8 (0.4)**	-3.0 (0.4)**	-0.4 (0.0)**	-0.2 (0.0)**
Heart Attack	1	-1.1 (0.3)*	-0.2 (0.2)	-1.7 (0.2)**	0.7 (0.3)*	-1.9 (0.3)**	-0.3 (0.0)**	-0.3 (0.0)**
	2	-3.0 (0.8)**	-2.8 (0.4)**	-1.3 (0.2)**	1.4 (0.4)*	-0.1 (0.9)	-0.1 (0.0)**	-0.3 (0.1)**
	3	-0.3 (0.4)	0.1 (0.2)	-0.5 (0.2)*	0.1 (0.3)	-0.7 (0.3)*	-0.0 (0.0)	-0.1 (0.0)**
All Heart Disease	1	-12.2 (0.7)**	-5.3 (0.3)**	-8.5 (0.3)**	-2.1 (0.6)**	-8.0 (0.6)**	-1.3 (0.1)**	-0.6 (0.0)**
	2	-11.5 (0.6)**	-5.1 (0.3)**	-6.7 (0.3)**	-5.4 (0.5)**	-5.8 (0.5)**	-0.9 (0.0)**	-0.3 (0.0)**
	3	-6.3 (0.6)**	-2.5 (0.3)**	-3.1 (0.3)**	-3.1 (0.5)**	-3.5 (0.5)**	-0.4 (0.0)**	-0.1 (0.0)*

[+] Models: 1 = unadjusted; 2 = adjusted for urbanicity, income inequality, % poverty, %< HS, %< college; 3 = Model 2 plus adjustment for % diabetes, % obesity, % hypertensive, % smokers. Abbreviations: WBI = well-being index; CLS = current life satisfaction; FLO = future life optimism

(* indicates P<0.05; ** indicates P<0.001)

© 2023 Spatz ES et al. *JAMA Network Open.*

eTable 3 Results of mediation analyses

The direct and indirect (through well-being) effects of Income inequality and ADI on total CVD mortality, and estimated mediation effect of the Well-Being Index*

	**Indirect Effects**			**Direct Effect**	**Total Effect (Direct+Indirect)**
	**SF->WBI**	**WBI->CVD mortality**	**SF->CVD mortality= (SF->WBI)*(WBI->CVD mortality)**	**SF on CVD mortality**	**SF->CVD mortality**
**Structural Factor (SF)**					
Gini (Income inequality)	-5.56 (0.56)	-0.08 (0.01)	0.43 (0.09)	6.90 (0.46)	7.32 (0.46)
ADI	-3.72 (0.48)	-0.02 (0.00)	0.08 (0.01)	2.93 (0.07)	3.01 (0.07)

*All effect sizes significant at the p<0.001 level

© 2023 Spatz ES et al. *JAMA Network Open.*
