# Supplementary material for: Association of Population Well-Being With Cardiovascular Outcomes
Source: JAMA Netw Open. 2023 Jul 5;6(7):e2321740. doi: 10.1001/jamanetworkopen.2023.21740 (PMC10323707; doi:10.1001/jamanetworkopen.2023.21740)
Supplement: Supplement 2. — Data Sharing Statement [file jamanetwopen-e2321740-s002.pdf]

Data Sharing Statement

Spatz. Association of Population Well-being With Cardiovascular Outcomes. *JAMA Netw Open*. Published July 05, 2023. doi:10.1001/jamanetworkopen.2023.21740

Data
Data available: Yes
Data types: Other (please specify)
Additional Information: We will provide deidentified population level data, aggregated at the county level
How to access data: Upon request, data will be provided. Please send requests to: jeph.herrin@yale.edu and erica.spatz@yale.edu
When available: beginning date: 03-10-2023

Supporting Documents
Document types: None

Additional Information
Who can access the data: Anyone requesting the data with a defined hypothesis
Types of analyses: Investigative research
Mechanisms of data availability: With investigator support
